# COVID-19-Related Concerns and Symptoms of Anxiety: Does Concern Play a Role in Predicting Severity and Risk?

**DOI:** 10.7759/cureus.19999

**Published:** 2021-11-29

**Authors:** Tarek Benzouak, Sasha Gunpat, Esther L Briner, Jennifer Thake, Steve Kisely, Sanjay Rao

**Affiliations:** 1 Department of Psychology, Carleton University, Ottawa, CAN; 2 Department of Psychiatry, University of Ottawa, Ottawa, CAN; 3 Department of Psychology, Concordia University, Montreal, CAN; 4 Department of Psychiatry, University of Queensland, Brisbane, AUS; 5 Departments of Psychiatry, Community Health & Epidemiology, Dalhousie University, Halifax, CAN

**Keywords:** pandemic, epidemiology, mental health, psychological stresses, anxiety, sars-cov-2, covid-19

## Abstract

Objective

Rates of anxiety have increased during the coronavirus disease 2019 (COVID-19) pandemic, partially attributable to the experience of COVID-19 related concerns. It remains pivotal to determine the implications of such concerns on the severity of anxiety as they may represent opportune targets for public health preventative or therapeutic efforts. The current study evaluated COVID-19 related concerns as predictors of anxiety symptom severity. It also assessed the relative risk associated with sub-types of COVID-19 concerns, the role of age, sex, and minority status as potential moderators; and the unique contribution of COVID-19 concerns beyond sociodemographics, perceived stress, and self-reported general mental health.

Methods

The data source was obtained from the publicly available ”Crowdsourcing: Impacts of COVID-19 on Canadians-Your Mental Health survey” conducted by Statistics Canada. Participants were Canadians aged 15 and older living in ten provinces or three territories. Only participants that completed the self-reported sociodemographics, COVID-19 concerns, and general anxiety symptoms measures were included (n = 44549). Multivariate linear regression was used to evaluate continuous reports of anxiety symptoms, and the relative risk of meeting anxiety cut-off levels was determined using chi-square non-parametric testing.

Results

Within the sample, 29.1% met cut-off levels of anxiety. Levels of coping and security (R^2^ = 0.205, p < 0.001), distal (R^2^ = 0.043, p < 0.001), and proximal concerns (R^2^ = 0.122, p < 0.001) were found to predict the severity of anxiety experiences, which was determined to be robust to statistical control for sociodemographics, perceived stress and self-reported general mental health (ΔR^2^ = 0.0625, p < 0.001). Minority status and sex were significant moderating variables, although the interaction accounted for less than 0.1% of the observed variance. Family stress from confinement, support during and after the crisis and personal health concerns significantly predicted more than a 200% increase in the risk of meeting anxiety cut-off levels.

Conclusion

The current study represents a novel examination of COVID-19-related concerns as risk factors for the experience of anxiety amongst a sizeable Canadian cohort. Coping and security-related concerns represented robust predictors of anxiety symptom experiences. Participants who experienced concerns relating to their proximal social groups were two times more at risk for meeting cut-off anxiety levels than individuals without such concerns. Longitudinal and evidence synthesis remains essential for identifying therapeutic targets and developing pandemic-related public health prevention and care.

## Introduction

Coronavirus disease 2019 (COVID-19) and the associated public health response, economic challenges, and uncertainty have had deteriorating impacts on mental health, including increases in the incidence of anxiety [1-3]. A national-level survey in Belgium showed an 8% increase in anxiety disorders since the beginning of the pandemic, while research in China revealed that many are worried about family members contracting the virus, with some reporting moderate to severe anxiety symptoms [4,5]. In another study from the United States, COVID-19 related stress predicted at least 30% of the variance in anxiety symptoms [6]. Previous research on COVID-19 has provided clues for those variables linked with anxiety, such as lockdowns, quarantine efforts, and other restrictions on daily life that may help fuel anxiety symptoms, such as worry and fear [7]. However, these factors have generally been evaluated using relatively small sample sizes. Quarantine is an unpleasant experience for many individuals because of separation from family and friends, feelings of boredom, fears of prolonged quarantine duration, financial instability, and uncertainty about disease status [8]. These consequences can impact mental health and promote experiences of anxiety.

Despite the increased risk of developing or worsening anxiety symptoms during the pandemic, there is a dearth of research identifying variables linked to the intensity of anxiety within a Canadian context. Analysis of these risk factors can further public health understanding of pandemic-related anxiety and guide the development of treatment strategies. The present study set out to address the link between COVID-19 related concerns and the incidence and intensity of anxiety symptoms. Of particular interest was exploring the moderating role of sociodemographics in the link between COVID-19 related concerns and anxiety.

## Materials and methods

Data source 

Data were obtained from the following web-only data longitudinal collection series from Statistics Canada ‘Crowdsourcing: Impacts of COVID-19 on Canadians’[9]. This data source is publicly available for analysis through Carleton University. Cross-sectional data were analyzed from the second iteration of crowdsourcing cycles, “Crowdsourcing: Impacts of COVID-19 on Canadians - Your Mental Health”, established to determine how Canadians are reacting to the COVID-19 crisis and the impact on their mental health. The target population was a convenience sample of Canadians aged 15 and older living in ten provinces or three territories. Participation in this initiative was voluntary. Prompts to participate were done through social media and various outside partners like other government agencies, private and public organizations, associations, and news channels. Data were collected directly from participants via a self-administered online questionnaire on demographic characteristics, mental health impacts, health behaviours, and COVID-19-related concerns. Data collection was available in English and French, and the questionnaire took approximately five minutes to complete. The data collection period started on April 24, 2020, and closed on May 11, 2020 (Appendix Tables).

Eligibility criteria

To be included in the analyses, participants had to complete the measures of COVID-19-related concerns and anxiety. As a result of missing data, 1440 participants were excluded from the current study, resulting in a final sample of 44549 participants.

Measures

COVID-19-Related Concerns

COVID-19-related concerns were assessed via 11 items from the Crowdsourcing: Impact of COVID-19 on Canadians - Your Mental Health survey (S-1). For each item, participants were asked to reflect on how they felt at the time of data collection. Specifically, participants were asked about how concerned they were about each of the presented questions relating to the impacts of COVID-19. Responses used a 4-point Likert scale, and the total score was calculated by summing responses to all 11 items. Higher scores were interpreted as increased COVID-19-related concerns.

Anxiety Symptoms

Anxiety was measured via seven items from the Generalised Anxiety Disorder (GAD-7) scale [10] which enquires about symptoms in the previous two weeks. Scores ranged from 0-21. Participants with a score of 10 or higher were considered to have moderate to severe symptoms of anxiety. Applying cut-off levels of 10, the identified specificity and selectivity for this scale have been reported to be 82% and 89%, respectively, and a good level of agreement between self-reported forms of the GAD-7 and interview-based administration of this scale has further been determined [10].

Demographic, Perceived Stress and General Mental Health.

Demographic moderators considered included age clusters (i.e., 15 to 24, 25 to 34, 35 to 44, 45 to 54, 55 to 64, 65 years and older), biological sex, and visible minority status. Visible minority status was operationalized as self-identification as South Asian, Chinese, Black, Filipino, Latin American, Arab, Southeast Asian, Korean, Japanese or any other ethnicity other than Caucasian or Indigenous. Participants were further asked to report their levels of perceived stress, general mental health, and perception of change in mental health levels across two-time points: before and after social restrictions were introduced.

Analysis plan

The study protocol was registered on Open Science Framework (https://osf.io/ahnbt). All analyses were conducted in SPSS version 27, and the alpha level was set at 0.05. Data were checked for impossible values, error codes, assumptions of linearity, multicollinearity, and normality before analyses. Analyses were conducted using case-wise deletion, resulting in an attrition rate of 3.13%. To assess for specific types of COVID-19-related concerns, an exploratory factor analysis (EFA) was conducted to identify the factor load. Multivariate linear regression and chi-square were conducted to assess if: (1) COVID-19-related concerns would predict anxiety symptom severity; (2) types of COVID-19 concerns differed in their association with anxiety symptoms; (3) age, sex, and visible minority status moderate the association between COVID-19-related concerns and anxiety symptoms; (4) COVID-19-related concerns contributed to anxiety beyond sociodemographics, perceived stress, and self-reported general mental health. To examine the variance in anxiety accounted for by COVID-19-related concerns, respective two-step linear regression was conducted for each identified COVID-19-related concern factor. A three-step model was utilized to determine the variance accounted for by COVID-19 concerns controlling for sociodemographics, perceived stress, and reports of general mental health. Following this, groups were formed using low and high reports of COVID-19-related concerns, which were cross tabbed with the cut-off score for anxiety to determine the relative risk associated with each COVID-19 related concern.

Finally, three separate exploratory linear regression analyses were conducted, adjusting for socio-demographics. They examined the moderating effect of age, sex, and visible minority status on the association between the total score of COVID-19 related concerns and anxiety symptoms. Unstandardized betas were reported for all regression analyses. Simple slope analysis and visualization were further conducted on all significant interactions using R (version 4.0.3) within the Rstudio environment (version 1.3.1073).

## Results

Participants (n = 44549) reported an average score of 26.43 (SD = 5.75) on the scale measuring COVID-19-related concerns, an average score of 7.13 (SD = 5.51) on the GAD-7, and 29.1 % met the cut-point for anxiety disorder. Sample descriptive statistics are presented in Table 1.

**Table 1:** Descriptive characteristics. Note: n = frequency.

Variable	n(%)
Age, years	
15 to 24	2271(5.1)
25 to 34	9814(22.0)
35 to 44	12081(27.1)
45 to 54	8746(19.6)
55 to 64	7103(15.9)
65 and older	4579(10.3)
Biological sex	
Male	11166(25)
Female	33428(75)
Visible Minority status	
Non-Minority	39972(90.6)
Visible Minority	4125(9.4)
Providence of residence	
Newfoundland and Labrador	532(1.2)
Prince Edward Island	202(0.5)
Nova Scotia	3028(6.8)
New Brunswick	923(2.1)
Quebec	5410(12.1)
Ontario	21446(48.1)
Manitoba	1316(3.0)
Saskatchewan	1133(2.5)
Alberta	3816(8.6)
British Columbia	6458(14.5)
Territories	330(0.7)
Rural/Urban residence	
Rural	5273(11.8)
Urban	38737(86.9)
Not stated	584(1.3)

COVID-19 concern factors

We conducted an EFA-with Principal Component Analysis using an orthogonal rotation (i.e., Varimax)-on the factor structure of the 11-item COVID-19-related concern scale [9]. The Kaiser-Meyer Olkin (KMO) measure verified the sampling adequacy for the analysis at KMO = 0.82, deemed as an excellent value to proceed with EFA [11]. The Bartlett’s Test of Sphericity was met at χ2 (55) = 176990.05, p < 0.001.

The analysis yielded three factors with eigenvalues over the Kaiser criterion [12] of 1, explaining a total of 62.796% of the variance for the entire set of variables. Visual examination of the scree plot justified retaining three factors. Factor labelled "coping and security" contained the following items: able to cope/support during crisis, support after the crisis, maintaining social ties, family stress from confinement, and civil disorder. This first factor (eigenvalue = 4.12) explained 37.49% of the variance. Factor 2 labelled "distal concerns" contained the following items: world population health, Canadian population health, and overloading health system. This second factor (eigenvalue = 1.65) explained 14.99% of the variance. Factor 3 labelled "proximal concerns" consisted of the following items: a member of household's health, my own health, and vulnerable people's health. This third factor (eigenvalue = 1.14) explained 10.32% of the variance.

All commonalities for these items were adequately related for factor analysis except one item, civil disorder, which demonstrated a small amount of variance (36.3%). Given that the low commonality of this variable was indicative of contributing only weakly with the other variables in the analysis, it was dropped from the scale, and a second EFA was computed. The KMO measure was still acceptable (KMO = 0.80), with Bartlett's Test of Sphericity significant at χ2 (45) = 164928.95, p < 0.001 in the second-factor analysis. Findings of the final model revealed that all items loaded onto three factors (S-2). Three clear patterns of COVID-19-related concerns were identified and independent of one another: "coping and security", "distal concerns", and "proximal concerns", used in the proceeding analysis.

Anxiety and COVID-19 concern clusters

**Figure 1:**
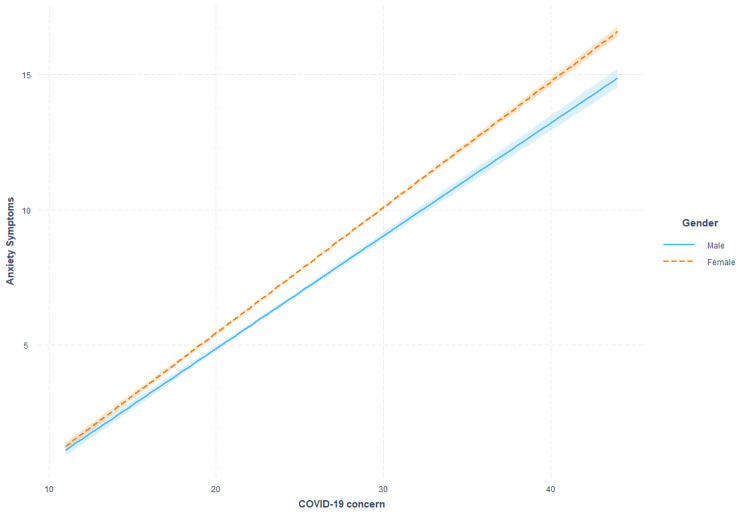
Moderating effects of sex on the association between COVID-19 concerns and anxiety symptoms.

**Figure 2:**
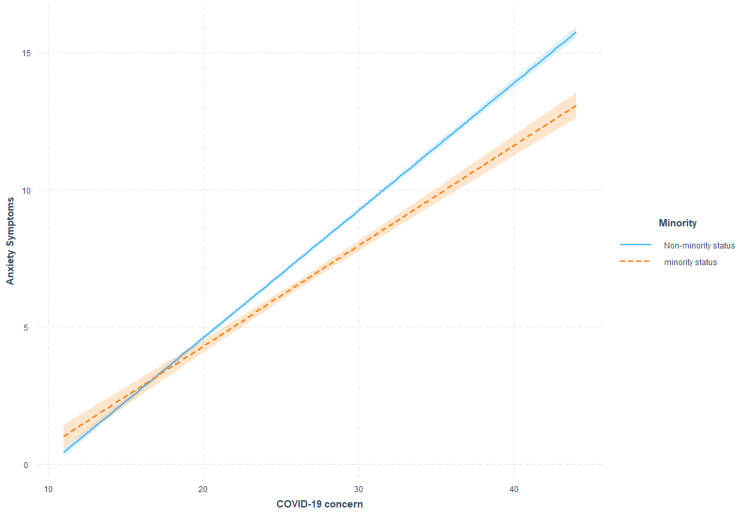
Moderating effects of visible minority status on the association between COVID-19 concerns and anxiety symptoms.

After adjusting for demographic factors, coping and security accounted for 20.5% of the variance in anxiety symptoms, this was followed by proximal concerns (12.2%) and distal concerns (4.3%; Table 2). After adjusting for demographic factors, age did not moderate the association between COVID-19 concerns and anxiety symptoms. However, sex interacted with COVID-19 concerns to predict less than 0.001% of the variance in anxiety symptoms. Both females (*B* = 0.46473, 95%CI[0.45537, 0.47409], SE = 0.00477, t = 97.35789, p < 0.001) and males (*B* = 0.41693, 95%CI[0.40078, 0.43308], SE = 0.00824, t = 50.59423, p < 0.001) were determined to experience anxiety symptoms as a function of COVID-19-related concerns, however females experienced greater levels of anxiety than males when considering high levels of COVID-19-related concerns (Figure 1). Additionally, minority status interacted with COVID-19 concerns to predict 0.01% of the variance in anxiety symptoms. Although both visible (*B* = 0.36576, 95%CI [0.34057, 0.39096], SE = 0.01285, t = 28.45373, p < 0.001) and non-visible minority (*B* = 0.46398, 95%CI [0.45541, 0.47255], SE = 0.00437, t = 106.11514, p < 0.001) groups were determined to experience an association between COVID-19-related concerns and anxiety symptoms, higher levels of COVID-19 concerns were more greatly linked to anxiety experiences for the non-minority group (Figure 2).

**Table 2:** Regression coefficients for associations between age, sex, minority status, COVID-19 concerns, and anxiety symptoms.

Predictor variable	B(95%CI)	SE	p
Anxiety and COVID-19 clusters			
Concern pandemic coping and security	3.61(3.55, 3.68)	0.032	<0.001
Concern distal concerns	1.62(1.55, 1.69)	0.035	<0.001
Concern proximal concerns	2.87(2.80, 2.94)	0.035	<0.001
Interaction terms			
Age x COVID-19 concerns	0.004(-0.002, 0.009)	0.003	0.187
Sex x COVID-19 concerns	0.048(0.030, 0.065)	0.009	<0.001
Minority status x COVID-19 concerns	-0.098(-0.122, -0.074)	0.012	<0.001

Relative risk of moderate to severe anxiety symptoms 

Individuals with a high level of COVID-19 concerns had higher risks of experiencing moderate to high levels of anxiety symptoms (Table 3). Although this effect was determined to be present for all types of COVID-19-related concerns, four specific types of concerns (i.e., family stress from confinement, support during and after the crisis, and personal health) were determined to be associated with a greater than 200% increased risk of reporting equal or above cut-off levels of anxiety symptoms.

**Table 3:** Relative Risk of anxiety report for each evaluated concern type.

Specific COVID-19 concern	Relative risk	X^2^	p
Family stress from confinement	2.80	5232	<0.001
Support during the crisis	2.25	3183	<0.001
Support after the crisis	2.18	2926	<0.001
Personal Health	2.05	2396	<0.001
Maintaining social ties	2.00	2275	<0.001
Health of a household member	1.97	2119	<0.001
Health of vulnerable people	1.97	1072	<0.001
Civil disorder	1.89	1842	<0.001
Canada's population's health	1.70	1228	<0.001
Overloading the health system	1.59	816.3	<0.001
World's population's health	1.46	629.6	<0.001

Perceived stress, mental health and COVID-19-related concerns

Perceived stress was determined to predict 34.8% of the variance associated with anxiety symptoms (*B* = 3.506, 95%CI [3.461, 3.551], SE = 0.023, t = 154.098, p < 0.001). In addition, perceived stress accounted for 21.1% of the variance in perceived mental health (*B* = 0.521, 95%CI [0.512, 0.530], SE = 0.005, t = 109.170, p < 0.001) and 15.8% of perceptions of change when comparing mental health during pre-and-post social distancing (*B* = 0.358, 95%CI [0.350, 0.366], SE = 0.004, t = 91.482, p < 0.001). Perceived mental health was highly correlated with anxiety experiences, accounting for 36.2% of the observed variation in anxiety reports (*B* = 3.153, 95%CI [3.114, 3.192], SE = 0.02, t = 158.892, p < 0.001). Modelling controlling for sociodemographics and inclusive of perceived mental health, stress and COVID-19 related concerns accounted for 55.51% of the explained variance in anxiety, of which 6.25% was uniquely attributed to COVID-19 related concerns (ΔR^2^ = 0.0625, F(3, 44017) = 2061.937, p < 0.001). Within this model, coping and security was found to uniquely account for 3.17% of anxiety experiences (B = 1.621, 95%CI [1.564, 1.678], SE = 0.029, t = 55.974, p < 0.001), followed by proximal concerns (R^2^part = 0.01061, *B* = 0.990, 95%CI [0.930, 1.050], SE = 0.031, t = 32.377, p < 0.001), and distal COVID-19 related concerns (R^2^part = 0.0001, *B* = 0.109, 95%CI [0.054, 0.164], SE = 0.014, t = 3.889, p < 0.001). Although perceived stress and COVID-19 related concerns were found to synergistically interact, the moderation of perceived stress on the association between COVID-19 related concerns and anxiety symptoms was determined to account for less than 0.6% of the variance in anxiety experiences (ΔR^2^ = 0.006, F(1, 44535) = 484.273, p < 0.001).

## Discussion

To our knowledge, there has been no previous study examining COVID-19-related concerns experienced by over 40 thousand Canadians at a national level. A recent meta-analysis has determined that the prevalence of anxiety within the general population is between 24% and 30% during the COVID-19 pandemic [13], comparable to 29.1 % of the sample meeting cut-off levels of the general anxiety assessment. As anxiety rates are estimated to be 7.3% during non-pandemic times [14], this study adds to the evidence that anxiety symptoms have increased during the pandemic.

Coping and security-related concerns were most predictive of heightened anxiety experiences, accounting for more than one-fifth of the observed variance in anxiety experiences. There could be a role for psychological interventions. A recent meta-analysis reported a meaningful reduction in anxiety symptoms during the COVID-19 pandemic as an outcome of a mixed and multicomponent self-guided intervention based on Cognitive Behavioural Therapy (CBT) and Acceptance and Commitment Therapy [15]. The feasibility and cost-effectiveness of such self-guided interventions may serve as a health promotion tool within the general population. As there is robust evidence demonstrating comparable efficacy of internet-based CBT and conventional in-person CBT [16]. Considering positive results in reducing anxiety symptoms reported in a recent pilot study examining internet-delivered CBT [17] and the current study's findings, there is ample justification for initiating open and randomized trials targeting factors such as experiences of COVID-19-related concerns. The development of accessible evidence-based interventions to mitigate the mental health impact of the pandemic is highly desirable.

Within the current sample, concerns relating to proximal social groups (e.g., family risk of infection) and vulnerable individuals were associated with greater anxiety symptoms when compared to concerns about distal populations such as Canadian or international population health. Furthermore, all pandemic-related concerns about social networks and personal health doubled the relative risk of meeting general anxiety symptoms cut-off levels, demonstrating a clear link between perceived proximal social hindrance and anxiety outcomes. The current evidence demonstrates that worries relating to one's social support network represent a key target to consider for the mitigation of COVID-19-related anxiety experiences. Social support has been a vastly accepted resource for coping with a stressor, serving a critical role in secondary stress appraisal [18]. Perceived social support has been demonstrated to moderate the longitudinal association between experienced loneliness and COVID-19-related anxiety, serving a protective role from anxiogenic outcomes within a relatively small Chinese community sample [19]. Notably, existing evidence has demonstrated that although not as effective as in-person interactions, web-based video communication may serve as a tool for health promotion. For instance, an open trial demonstrated that five online chat sessions with an anonymous partner increase self-esteem and social support perceptions while decreasing loneliness and depressive symptoms [20]. However, within the context of COVID-19, the utility of web-based video communication as a mitigating factor has received minimal attention. As the general public becomes more competent with technology usage, computer-mediated remote interactions may be a valuable mitigation tool for promoting social support when face-to-face support opportunities are diminished.

Although higher reports of distal COVID-19 concerns predicted greater anxiety symptoms, the observed effect size for this concern was low compared to coping, personal, and proximal health concerns. Future public health communication relating to COVID-19 concerns can benefit from such findings. The results from the current study point toward potential benefits from the provision of information on mitigation strategies specific to coping and the health of proximal social groups, as opposed to emphasizing global or distal information. In addition, there have been various lines of evidence demonstrating benefits from tailored health communication approaches [21].

The associations between COVID-19-related concerns and anxiety experiences were not found to be influenced by age. However, the association between COVID-19-related concerns and anxiety was greater in females and individuals identifying as not part of a visible minority in the circumstance the COVID-19-related concerns were severe. These findings align with previous Canadian evidence examining the role of minority status [22] and gender [23] in the context of anxiety experiences. However, our findings suggest that lower rates of anxiety within non-minority and male groups are only observed under circumstances in which perceived stressors, such as COVID-19-related concerns, are high. However, it is essential to note that the variance associated with these interactions was determined to be smaller than 0.1% in both cases, suggesting minimal value in targeting specific sex or minority identities in the context of reducing the implications of COVID-19 concerns on anxiety outcomes. 

The current evidence further demonstrates that perceived stress and self-reports of mental health are major risk factors for the experience of anxiety during the pandemic. However, COVID-19-related concerns were determined to account for anxiety symptoms beyond the current sample's perceptions of stress and general mental health. The implications of COVID-19 may thus operate to some degree outside the implications of psychopathology through the modulation of perceptions of well-being.

Higher COVID-19-related concerns might uniquely increase anxiety in a few ways, including increases in maladaptive thinking and decision making [24], as well as reducing involvement in well-being protective factors, such as engagement in meaningful activities [25]. Interestingly, research has revealed that those who report greater meaning or purpose in life seemed to be less anxious [26] and more compliant with public health guidelines [27]. Thus, decreases in well-being may result in greater experienced COVID-19 concerns due to greater engagement in non-protective behaviours. However, there remains a need for longitudinal evaluations of the potential mechanistic implications of well-being experiences on the association between COVID-19 related concerns and severity levels of experienced anxiety within the general public.

Limitations

The current study is highly powered due to the analyses of a large sample, allowing the precision necessary to detect potential minor effects relating to specific concern types. Furthermore, the current study included participants from all territories and provinces in Canada. However, the study includes several limitations to consider when interpreting the current findings. First and foremost, data analyses were cross-sectional and thus did not account for temporal factors; participants with high anxiety may have had high anxiety prior to the COVID-19 outbreak. In addition, although the current sample was derived from the entire Canadian landscape, it does not entail national representativeness. Although the GAD-7 has been repeatedly demonstrated to be reliable and valid, the assessment tool for COVID-19-related concerns has not undergone rigorous validity assessments as it was created for the intent of this data collection. Assessments of participant perceptions of stress and general mental health were limited due to a single item assessment of these experiences. Regarding ethnic differences, the current study only had access to a dichotomized visible minority status. However, evidence has demonstrated that anxiety experiences differ within ethnic minorities; for instance, South Asians have reported lower anxiety symptoms than African Canadians [22]. Similarly, evidence has demonstrated that financial challenges are significant predictors of anxiety during the COVID-19 pandemic [28]; however, data relating to financial experiences were not available for analysis. Thus, these distinctions were not possible within the current study but remain essential for the scientific understanding of experienced concerns and anxiety within the context of the COVID-19 pandemic.

## Conclusions

The current study represents a novel examination of COVID-19-related concerns as risk factors for the experience of anxiety in the context of both dichotomized cut-off and continuous symptom severity amongst a sizeable Canadian cohort. The results indicate that COVID-19 related concerns represent robust predictors of anxiety experiences during the COVID-19 pandemic. The experience of coping and security-related concerns were found to be stronger predictors than distal social concerns. In addition, participants who reported experiencing concerns relating to their proximal social groups had twice the risk of meeting anxiety cut-off levels when compared to individuals without such concerns. 

Although it is vastly accepted that worry derived from experiences of concerns serves as a predisposing factor for anxiety experiences, future studies examining longitudinal associations between these factors within a Canadian context remain unaddressed. Given that COVID-19 restrictions and policy vary from one province or territory to another, further examination of a representative sample for each province and territory may provide data to compare COVID-19 restriction implications on the association between COVID-19 concerns and anxiety outcomes. Further, developing a psychometrically validated tool to examine pandemic-related implications is primordial for optimizing the validity of data collection in the context of future pandemics. One potential solution may be achieved through the psychometric development of a non-specific general pandemic questionnaire that can be administered within a vast spectrum of communicable outbreaks.

With the increased inoculation for SARS-CoV-2 infection, the window for data collection relating to the pandemic may be closing. Hence, longitudinal and evidence synthesis remains essential for identifying therapeutic targets and developing pandemic-related public health prevention and care. 

## Appendices

**Table 4:** Statistics Canada’s COVID-19 concerns assessment tool. Response options for all questions relating to COVID-19 concerns ranged from: Not at all (1), Somewhat (2), Very (3), and Extremely (4). Reference: Statistics Canada (2020). ICC-MH 2020 - Data Dictionary. Statistics Canada. Public Use Microdata File Impacts of COVID-19 on Canadians - Mental Health Crowdsource file Public Use Microdata File. DOI: https://doi.org/10.25318/13250002-eng.

COVID-19-related concern	Question
My own health	How concerned are you about each of the following impacts of COVID-19? - My own health
Member of the household’s health	How concerned are you about each of the following impacts of COVID-19? - Member of the household’s health
Vulnerable people’s health	How concerned are you about each of the following impacts of COVID-19? - Vulnerable people’s health
Canadian population’s health	How concerned are you about each of the following impacts of COVID-19? - Canadian population’s health
World population’s health	How concerned are you about each of the following impacts of COVID-19? - World population’s health
Overloading the health system	How concerned are you about each of the following impacts of COVID-19? - Overloading the health system
Civil disorder	How concerned are you about each of the following impacts of COVID-19? - Civil disorder
Maintaining social ties	How concerned are you about each of the following impacts of COVID-19? - Maintaining social ties
Ability to cooperate and support one another during the crisis	How concerned are you about each of the following impacts of COVID-19? - Ability to cooperate and support one another during the crisis
Ability to cooperate and support one another after the crisis	How concerned are you about each of the following impacts of COVID-19? - Ability to cooperate and support one another after the crisis
Family stress from confinement	How concerned are you about each of the following impacts of COVID-19? - Family stress from confinement

**Table 5:** Final factor loadings for items on the COVID-19-related concerns scale.

Item	Loadings			
	Factor 1: Concern 1	Factor 2: Concern 2	Factor 3: Concern 3	Communality
					Able coop/supp during crisis	0.836			0.754
					Able coop/supp after crisis	0.8			0.698
Maintaining social ties	0.779			0.614
Family stress from confinement	0.622			0.484
World population's health		0.887		0.798
Canadian population's health		0.868		0.818
Overloading the health system		0.684		0.557
Member of household's health			0.856	0.763
My own health			0.765	0.634
Vulnerable people's health			0.531	0.501
Eigenvalue	3.86	1.63	1.13	
% of Total Variance	38.62	16.32	11.28	
Total Variance			66.22%	
